# Frequency, type, and distribution of EST-SSRs from three genotypes of *Lolium perenne*, and their conservation across orthologous sequences of *Festuca arundinacea, Brachypodium distachyon*, and *Oryza sativa*

**DOI:** 10.1186/1471-2229-7-36

**Published:** 2007-07-12

**Authors:** Torben Asp, Ursula K Frei, Thomas Didion, Klaus K Nielsen, Thomas Lübberstedt

**Affiliations:** 1 Department of Genetics and Biotechnology, University of Århus, Research Centre Flakkebjerg, Forsøgsvej 1, 4200 Slagelse, Denmark; 2 DLF-Trifolium Ltd., Research Division, 4660 Store Heddinge, Denmark

## Abstract

**Background:**

Simple sequence repeat (SSR) markers are highly informative and widely used for genetic and breeding studies in several plant species. They are used for cultivar identification, variety protection, as anchor markers in genetic mapping, and in marker-assisted breeding. Currently, a limited number of SSR markers are publicly available for perennial ryegrass (*Lolium perenne*). We report on the exploitation of a comprehensive EST collection in *L. perenne *for SSR identification. The objectives of this study were 1) to analyse the frequency, type, and distribution of SSR motifs in ESTs derived from three genotypes of *L. perenne*, 2) to perform a comparative analysis of SSR motif polymorphisms between allelic sequences, 3) to conduct a comparative analysis of SSR motif polymorphisms between orthologous sequences of *L. perenne*, *Festuca arundinacea, Brachypodium distachyon*, and *O. sativa*, 4) to identify functionally associated EST-SSR markers for application in comparative genomics and breeding.

**Results:**

From 25,744 ESTs, representing 8.53 megabases of nucleotide information from three genotypes of *L. perenne*, 1,458 ESTs (5.7%) contained one or more SSRs. Of these SSRs, 955 (3.7%) were non-redundant. Tri-nucleotide repeats were the most abundant type of repeats followed by di- and tetra-nucleotide repeats. The EST-SSRs from the three genotypes were analysed for allelic- and/or genotypic SSR motif polymorphisms. Most of the SSR motifs (97.7%) showed no polymorphisms, whereas 22 EST-SSRs showed allelic- and/or genotypic polymorphisms. All polymorphisms identified were changes in the number of repeat units. Comparative analysis of the *L. perenne *EST-SSRs with sequences of *Festuca arundinacea*, *Brachypodium distachyon*, and *Oryza sativa *identified 19 clusters of orthologous sequences between these four species. Analysis of the clusters showed that the SSR motif generally is conserved in the closely related species *F. arundinacea*, but often differs in length of the SSR motif. In contrast, SSR motifs are often lost in the more distant related species *B. distachyon *and *O. sativa*.

**Conclusion:**

The results indicate that the *L. perenne *EST-SSR markers are a valuable resource for genetic mapping, as well as evaluation of co-location between QTLs and functionally associated markers.

## Background

*Lolium perenne *is one of the major grass species used for turf and forage in the temperate regions of the world. It belongs to the grass family *Poaceae*. *L. perenne *(2n = 2x = 14) is taxonomically related to many important plant species in the *Poaceae *family, including rice (*Oryza sativa*), wheat (*Triticum aestivum *L.), barley (*Hordeum vulgare *L.), maize (*Zea mays *L.), and sorghum (*Sorgum bicolor *L.) [1].

Several anonymous molecular markers have been developed for *L. perenne*, including restriction fragment length polymorphism and random amplified polymorphic DNA [2,3], amplified fragment length polymorphism [4], as well as SSR markers [5,6]. More recently, gene-tagged markers [7] have been developed and used to construct genetic linkage maps [8-10]. Although there have been several reports on *L. perenne *SSR marker development, most of these markers are currently not publicly available [8,9]. Furthermore, synteny to other *Poaceae *species is based on a limited number of anchor markers [11], reinforcing the need for more publicly available gene-derived EST-SSR markers for *L. perenne*.

Simple sequence repeats (SSRs) have become one of the most widely used molecular marker systems in plant genetics and breeding. They are widely used for genetic diversity assessment, variety protection, molecular mapping, and marker assisted selection, providing an efficient tool to link phenotypic and genotypic variation [12-14].

SSRs are tandem repeated sequences comprised of mono-, di-, tri-, tetra-, penta-, or hexa-nucleotide units [15,16]. SSRs are ubiquitous in prokaryotes and eukaryotes and can be found both in coding- and non-coding regions. They are ideal as molecular markers because of the co-dominant inheritance, relative abundance, multi-allelic nature, extensive genome coverage, high reproducibility, and simple detection [12].

The number of SSR motifs at a locus is variable, because SSRs experience a high rate of reversible length-altering mutations by unequal crossing over and replication slippage, where the transient dissociation of the replicating DNA strand is followed by misaligned re-association [17,18]. SSRs are among the most variable DNA sequences in the genome [19], and the mutation rate and type depends mainly on the number of repeat motifs [20]. However, the mutation rates differ among loci and among alleles, and also between species [21]. The resulting mutations, which typically add or subtract one or a few repeat motifs, can be reversed by a subsequent mutation at the same or any other point in the repeat motif [22]. In addition, point mutations in a repeat motif may result in an imperfect repeat motif, that in turn can be eliminated and converted back to a perfect motif again by replication slippage, which tends to eliminate imperfect repeats [22].

Whereas earlier studies on SSR marker development primarily utilized anonymous DNA fragments containing SSRs isolated from genomic libraries, more recent studies have used computational methods to detect SSRs in sequence data generated from large-scale EST sequencing projects. About 1 to 5% of ESTs from different plant species have been found to contain SSRs suitable for marker development [23]. EST-SSR markers have been developed for a number of plant species, including grape [24], rice [25], durum wheat [26], rye [27], barley [28], barrel medic [29], ryegrass [8], wheat [30], and cotton [31]. EST-SSR markers are gene-tagged markers directly associated with an expressed gene and, thus, completely linked with putative qualitative or quantitative trait locus alleles. EST-SSR markers are, therefore, superior and more informative compared to anonymous markers [7].

The conservation of grass genomes has been comprehensively documented, and comparative genomics has become an important strategy to extend genetic information from model species to species with a more complex genome, as well as between related species with complex genomes [11,32]. As EST-SSR markers are derived from expressed genes, they are more conserved and have a higher level of transferability to related species than anonymous DNA markers. They are, therefore, useful as anchor markers for comparative mapping across species, comparative genomics, and evolutionary studies [23,24,28,29,33,34]. However, the conserved nature of EST-SSRs may also limit their degree of polymorphism. The transferability of SSR loci across species within a genus has in several studies been above 50% [28,29,35-37], whereas the transferability of SSR loci across genera was poor [28,35,38,39].

We report on the exploitation of a comprehensive EST collection in *L. perenne *for SSR identification. The objectives of this study were 1) to analyse the frequency, type, and distribution of SSR motifs in ESTs derived from three genotypes of *L. perenne*, 2) to perform a comparative analysis of SSR motif polymorphisms between allelic sequences, 3) to conduct a comparative analysis of SSR motif polymorphisms between orthologous sequences of *L. perenne*, *Festuca arundinacea, Brachypodium distachyon*, and *O. sativa *4) to identify functionally associated EST-SSR markers for application in comparative genomics and breeding.

## Results

### Identification and characterization of EST-SSRs

A total of 31,379 single-pass sequencing reactions on random *L. perenne *cDNA clones from 13 cDNA libraries resulted in 25,744 high-quality ESTs (Table 1). Of these ESTs, 9,177 (3.85 Mb) were derived from the genotype NV#20F1-30, 4,394 (1.75Mb) from the genotype NV#20F1-39, and 12,173 (8,53 Mb) from the genotype F6 (Table 2). The 25,744 ESTs assembled into 3,195 tentative consensus sequences and 6,170 singletons, thus representing 9,365 unique sequences.

**Table 1:** Plant material used for cDNA library construction in *Lolium perenne*, and number of reads from each cDNA library.

**cDNA library name**	**Plant material**	**Genotype**	**Number of reads**	**Number of Phred ≥ 20 reads**
rg1	Ethiolated leaves	NV#20F1-30	4,242	3,857
rg2	Leaves from nitrogen depleted plants	NV#20F1-39	346	322
rg3	Leaves from cold stressed plants	NV#20F1-39	4,069	3,546
rg4	Meristem	NV#20F1-39	325	307
rg5	Stem	NV#20F1-30	1,529	1,474
rg6	Leaves from drought stressed plants	NV#20F1-30	4,014	3,667
rg7	Senescing leaves	NV#20F1-30	330	303
r	Root	F6	7,004	6,870
p	Pollen	F6	425	335
ve	Vegetative shoot	F6	2,999	2,842
vr	Vernalized shoot	F6	490	423
sa/sb	Seedling	F6	2,805	2,435
gsa/gsb	Germinating seeds	F6	2,801	2,519

**Table 2:** Summary of EST-SSR searches for the *Lolium perenne *genotypes NV#20F1-30, NV#20F1-39, and F6, and for the combined dataset.

	**NV#20F1-30**	**NV#20F1-39**	**F6**	**Combined**
Total number of sequences examined:	9,177	4,394	12,173	25,744
Total size of examined sequences (bp):	3,846,707	1,751,833	2,932,559	8,531,099
Total number of identified SSRs:	353	174	1,074	1,601
Number of SSR containing sequences:	327	161	970	1,458
Number of sequences containing more than 1 SSR:	25	13	95	133
Number of SSRs present in compound formation:	15	6	75	96
**Repeat types**				
Di-nucleotide type:	58	45	92	195
Tri-nucleotide type:	237	102	914	1,253
Tetra-nucleotide type:	54	25	47	126
Penta-nucleotide type:	4	1	13	18
Hexa-nucleotide type:	0	1	8	9
Number of ESTs per SSR:	26.0	25.3	11.3	16.1
Kb sequence per SSR:	10.9	10.1	2.7	5.3

The 25,744 ESTs from the three genotypes of *L. perenne *were screened for SSRs using the MISA software [28]. As shown in Table 2, a total of 1,458 redundant ESTs containing an SSR were identified from the 25,744 ESTs. Thus 5.66% ESTs contain at least one SSR. Cluster analysis of the EST-SSRs yielded a final number of 955 (3.71%) non-redundant EST-SSRs. The percentage of redundant ESTs containing an SSR of the two genotypes NV#20F1-30 and NV#20F1-39 was 3.56 and 3.66, respectively, whereas the percentage of ESTs containing an SSR of the genotype F6 was 9.97%. On average, approximately one SSR was found per 10 kb in the genotypes NV#20F1-30 and NV#20F1-39, whereas one SSR was found per 2.7 kb in the genotype F6, corresponding to a total of approximately 26 ESTs per SSR for the two genotypes NV#20F1-30 and NV#20F1-39, and 11 ESTs per SSR for the genotype F6. A total of 133 ESTs had more than one SSR motif, 96 of which were considered the compound type according to the predefined criteria (Table 2).

The occurrences of different repeat unit size SSRs of the ESTs from the NV#20F1-30 genotype were 16.4% di-, 67.1% tri-, 15.3% tetra-, and 1.1% penta-repeat units. For the NV#20F1-39 genotype the occurrences were 25.9% di-, 58.6% tri-, 14.4% tetra-, 0.6% penta-, and 0.6% hexa-repeat units, and for the F6 genotype the occurrences were 8.6% di-, 85.1% tri-, 4.4% tetra-, 1.2 % penta-, and 0.7% hexa-repeat units.

**Figure 1:**
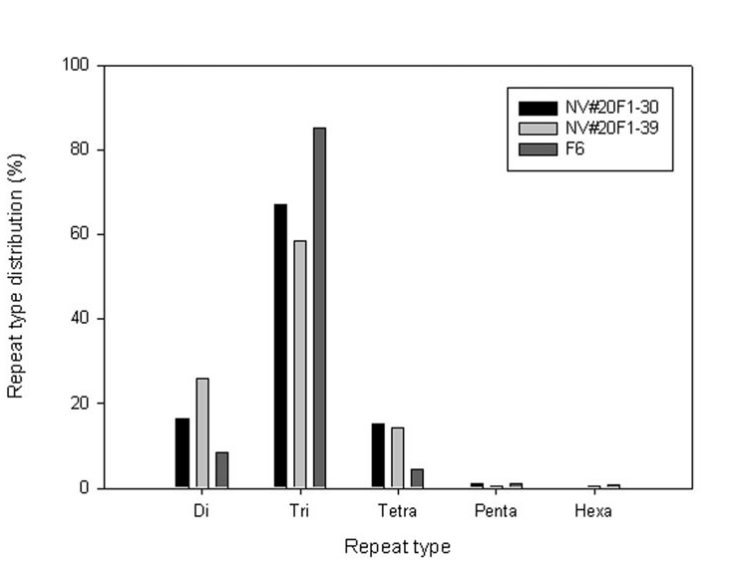
Distribution of different repeat type classes for EST-SSRs of the *Lolium perenne* genotypes NV#20F1-30, NV#20F1-39, and F6.

In the datasets from the genotypes NV#20F1-30 and F6, there were significantly (*X*^2^; p < 0.05) more tri-repeat than di- and tetra- repeat SSRs, while in the dataset from the genotype NV#20F1-39, there were significantly (*X*^2^; p < 0.05) more di- and tri- than tetra- repeat SSRs (Figure 1). No significant differences (*X*^2^; p < 0.05) was observed between genotypes with respect to tri- and tetra- repeat SSRs, while the EST-SSRs derived from the genotype NV#20F1-39 contained significantly (*X*^2^; p < 0.05) more di-repeat SSRs compared to the EST-SSRs derived from the other two genotypes. The frequency of the SSR motifs (any two complementary sequences considered one motif) are listed in Table 3 for the EST-SSRs from NV#20F1-30, NV#20F1-39, and F6, and in Table 4 for the combined dataset.

**Table 3:** The frequency of different types of repeats in redundant EST-SSR from the genotypes NV#20F1-30, NV#20F1-39, and F6.

**Repeat motif**	**NV#20F1-30**			**NV#20F1-39**			**F6**		
	**Tetra**	**Penta**	**≥ Hexa**	**Tetra**	**Penta**	**≥ Hexa**	**Tetra**	**Penta**	**≥ Hexa**

AC/GT	-	-	20	-	-	13	-	-	23
AG/CT	-	-	15	-	-	5	-	-	60
AT/AT	-	-	22	-	-	22	-	-	4
CG/CG	-	-	1	-	-	1	-	-	5
AAC/GTT	16	1		16			10	1	
AAG/CTT	26	6	3	13	2	1	43	17	6
AAT/ATT	19			6	2		8		1
ACC/GGT	14	3		4			43	10	9
ACG/CGT	13	2	1	7		1	51	7	1
ACT/AGT	13	3	1	1		1	7	2	
AGC/GCT	36	6	1	15	3	4	57	19	9
AGG/CCT	11	1		3			114	14	11
ATC/GAT	33	18	5	14	7		19	5	1
CCG/CGG	5			2			302	86	61
AAAG/CTTT	4		1					1	
AAGG/CCTT				2	1		6		
AATG/CATT	5			5			2		
ACGC/GCGT							1		
ACGG/CCGT									
ACGT/ACGT							1		
ACTC/GAGT	5			4			6		
AGAT/ATCT	18			4			5		
AGCC/GGCT							5		
AGCG/CGCT							1	1	
AGCT/AGCT				1			4		
AGGG/CCCT							6		
AGGT/ACCT									
CCCG/CGGG							2		
CCGG/CCGG				1			1		
CATC/GATG	2						1		
CTGC/GCAG							1		
GATC/GATC	7	1		1			2		
GCAT/ATGC							1		
AACC/GGTT	1	1							
AGTG/CACT				1					
ATAC/GTAT		2			1				
CCGA/TCGG	2			3					
GATG/CATC									
TATC/GATA			1						
TGTA/TACA		1	3		1				
AAGAG/CTCTT							1		
TCCCA/TCCCA							1		
TCGTC/GACGA							3		
AGAGG/CCTCT	1						2		
ATCGC/GCGAT							1		
CCGCT/AGCGG							1		
GCGAG/CTCGC							1		
TGTCG/CGACA							3		
CATGG/CCATG	1								
GATCT/AGATC	1								
GTGTT/AACAC				1					
TGTGG/CCACA	1								
AGAACA/TGTTCT									
ACCTCC/GGAGGT							1		
ACTCCT/AGGAGT								2	
AGAGGC/GCCTCT							1		
AGAGGG/CCCTCT								1	
AGAGGT/ACCTCT							1		
AGCTCC/GGAGCT							1		
GAAGAG/CTCTTC				1			1		

**Table 4:** The frequency of different types of repeats in redundant EST-SSRs from the three genotypes NV#20F1-30, NV#20F1-39, and F6.

**Repeat motif**	**Number of repeats**								**Total**	**%**
			
	**4**	**5**	**6**	**7**	**8**	**9**	**10**	**>10**		
AC/GT	-	-	31	9	7	6	1	2	56	3.50
AG/CT	-	-	33	19	9	5	12	6	84	5.25
AT/AT	-	-	32	13	3				48	3.00
CG/CG	-	-	3	2		2			7	0.44
AAC/GTT	42	2							44	2.75
AAG/CTT	82	25	9			1			117	7.31
AAT/ATT	33	2	1						36	2.25
ACC/GGT	61	13	6	2	1				83	5.18
ACG/CGT	71	9	2	1					83	5.18
ACT/AGT	21	5	2						28	1.75
AGC/GCT	104	26	9	1		1			141	8.81
AGG/CCT	128	15	5	1		2	3		154	9.62
ATC/GAT	70	32	3	4		1	1		111	6.93
CCG/CGG	309	86	32	15	7	5		2	456	28.48
AAAG/CTTT	4	1	1						6	0.37
AAGG/CCTT	8	1							9	0.56
AATG/CATT	12								12	0.75
ACGC/GCGT	1								1	0.06
ACGG/CCGT										
ACGT/ACGT	1								1	0.06
ACTC/GAGT	15								15	0.94
AGAT/ATCT	27								27	1.69
AGCC/GGCT	5								5	0.31
AGCG/CGCT	1	1							2	0.12
AGCT/AGCT	5								5	0.31
AGGG/CCCT	6								6	0.37
AGGT/ACCT										
CCCG/CGGG	2								2	0.12
CCGG/CCGG	2								2	0.12
CATC/GATG	3								3	0.19
CTGC/GCAG	1								1	0.06
GATC/GATC	9								9	0.56
GCAT/ATGC	1								1	0.06
AACC/GGTT	1	1							2	0.12
AGTG/CACT	1								1	0.06
ATAC/GTAT		3							3	0.19
CCGA/TCGG	5								5	0.31
GATG/CATC	1	1							2	0.12
TATC/GATA								1	1	0.06
TGTA/TACA		2		3					5	0.31
AAGAG/CTCTT	1								1	0.06
TCCCA/TCCCA	1								1	0.06
TCGTC/GACGA	3								3	0.19
AGAGG/CCTCT	3								3	0.19
ATCGC/GCGAT	1								1	0.06
CCGCT/AGCGG	1								1	0.06
GCGAG/CTCGC	1								1	0.06
TGTCG/CGACA	3								3	0.19
CATGG/CCATG	1								1	0.06
GATCT/AGATC	1								1	0.06
GTGTT/AACAC	1								1	0.06
TGTGG/CCACA	1								1	0.06
AGAACA/TGTTCT	1								1	0.06
ACCTCC/GGAGGT		2							2	0.12
ACTCCT/AGGAGT	1								1	0.06
AGAGGC/GCCTCT		1							1	0.06
AGAGGG/CCCTCT	1								1	0.06
AGAGGT/ACCTCT	1								1	0.06
AGCTCC/GGAGCT	1								1	0.06
GAAGAG/CTCTTC	1								1	0.06

In some cases, the frequency of SSR motifs for EST-SSRs varied significantly (*X*^2^; p < 0.05) between the three genotypes (Table 3). In the genotype F6, the SSR motif CCG/CGG was identified in 41.8% of the EST-SSRs but only in 1.4% and 1.2% of the respective EST-SSRs in the genotypes NV#20F1-30 and NV#20F1-39.

#### *In silico *analysis of allelic and genotypic SSR motif polymorphisms

A total of 521 contigs containing an SSR motif were identified from the 3,195 *L. perenne *contigs. The individual sequences within each contig were analysed for SSRs, and the results of the SSR searches were subsequently compared within each contig, to identify allelic- and/or genotypic polymorphisms at the SSR motif. A total of 22 contigs containing EST sequences with either allelic- and/or genotypic SSR polymorphisms were identified, corresponding to 2.3% of the non-redundant EST-SSR contigs (Table 5).

**Table 5:** Comparative analysis of EST-SSRs between the genotypes NV#20F1-30, NV#20F1-39, and F6.

	**NV#20F1-30**		**NV#20F1-39**		**F6**	
	
	**Allele 1**	**Allele 2**	**Allele 1**	**Allele 2**	**Allele 1**	**Allele 2**
Contig 0576	n.d.	n.d.	n.d.	n.d.	(TC)6ccctcgagtcgagtcctcccggcgagtctct (GCG)5	(TC)4ccctcgagtcgagtcctcccggcgagtctct (GCG)7
Contig 0395	n.d.	n.d.	n.d.	n.d.	(GCC)5	(GCC)4
Contig 0850	n.d.	n.d.	n.d.	n.d.	(GAG)10	(GAG)9
Contig 1068	n.d.	n.d.	n.d.	n.d.	(AGC)4	(AGC)5
Contig 2174	n.d.	n.d.	n.d.	n.d.	(CGC)7	(CGC)9
Contig 2043	n.d.	n.d.	n.d.	n.d.	(TGC)6	(TGC)4
Contig 0538	n.d.	n.d.	n.d.	n.d.	(GGT)4	(GGT)3
Contig 2873	n.d.	n.d.	n.d.	n.d.	(CCT)5	(CCT)4
Contig 2944	n.d.	n.d.	n.d.	n.d.	(GGC)4	(GGC)3
Contig 0131	n.d.	n.d.	n.d.	n.d.	(GGC)4	(GGC)3
Contig 0656	n.d.	n.d.	n.d.	n.d.	(GA)11tggcgtcggcagcaacggcgacgc (CGG)4	(GA)8tagagatggcgtcggcagcagcggcgacgc(CGG)4
Contig 3185	n.d.	n.d.	n.d.	n.d.	(CGC)5	(CGC)4
Contig 2810	n.d.	n.d.	n.d.	n.d.	(CCT)4tccctctcctctccccct (CGC)6	(CCT)4tccctctcccctccccct (CGC)5
Contig 2542	n.d.	n.d.	n.d.	n.d.	(CTC)4	(CTC)6
Contig 1034	n.d.	n.d.	n.d.	n.d.	(CGC)4	(CGC)5
Contig 3128	n.d.	n.d.	(GA)10	(GA)9	n.d.	n.d.
Contig 2765	(ATGC)4ctatgcatggatgtgtggaagctcctttgcatgtac(AT)6	(ATGC)4ctatgcatggatgtgtggaagctcctttgcatgtac(AT)8	n.d.	n.d.	n.d.	n.d.
Contig 0720	(CTG)5	(CTG)4	n.d.	n.d.	n.d.	n.d.
Contig 2888	(TGTA)7	n.d.	(TGTA)5	n.d.	n.d.	n.d.
Contig 0855	(TA)8	n.d.	(TA)7	n.d.	n.d.	n.d.
Contig 1520	(TGA)5	n.d.	(TGA)6	(TGA)7	n.d.	n.d.
Contig 0700	(ATG)5	n.d.	(ATG)4	n.d.	(ATG)5	n.d.

n.d: No allelic sequence present in the EST collection.

In all 22 contigs, the SSR motif polymorphisms identified were changes in the number of repeat units, while no contigs were identified with changes in the repeat type. Most of the SSR motif polymorphisms were one to two repeat unit changes, and the maximum number of repeat unit changes observed were three (Table 5).

A total number of two and one allelic SSR polymorphism were identified in contigs containing EST sequences derived from the genotype NV#20F1-30 and NV#20F1-39, respectively, while fifteen allelic SSR polymorphisms were identified in contigs containing EST sequences derived from the genotype F6 (Table 5). Comparing SSR motif polymorphisms between NV#20F1-30 and NV#20F1-39 identified two contigs containing genotypic SSR motif polymorphisms. Contig 1520 contains both genotypic and allelic SSR motif polymorphisms, with genotypic SSR motif polymorphism between the genotypes NV#20F1-30 and NV#20F1-39, as well as allelic SSR motif polymorphism between alleles derived from the genotype NV#20F1-39. Contig 0700 contains one allele from each of the three genotypes, with a genotypic SSR motif polymorphism in the allele derived from the genotype NV#20F1-39, while no genotypic SSR motif polymorphisms were identified in alleles derived from the other two genotypes (Table 5).

### *In silico *analysis of the conservation of SSR motifs between four species of the Poaceae family

Molecular markers designed to the transcribed region of the genome are often transferable among related species, because gene sequences remain highly conserved during evolution. Molecular markers designed to the transcribed region of the genome can thus be used to construct comparative genetic maps, facilitating the study of synteny conservation, and co-linearity among related genomes.

An *in silico *approach was used to validate the *L. perenne *EST-SSRs as molecular markers in comparative genetic studies. The non-redundant dataset of 955 *L. perenne *EST sequences containing an SSR, were blasted using BlastN (e-value 1.00E-10) against 41,834 *F. arundinacea *EST sequences, 3,818 *B. distachyon *contigs, and 32,132 full-length *O. sativa *cDNA sequences, to identify the orthologous sequences of these species. The blast searches resulted in 833, 540, and 26 orthologous sequences of *F. arundinacea, B. distachyon*, and *O. sativa*, respectively. A dataset of 19 clusters of sequences containing orthologous sequences from all four species was identified and aligned using ClustalW [40]. All alignments were analysed for SSR motif polymorphisms between the four species (Table 6).

**Table 6:** Comparative analysis of SSRs motif polymorphisms between *Lolium perenne, Festuca arundinacea, Brachypodium distachyon*, and *Oryza sativa*. The cross-species comparison of SSR motif polymorphisms was performed as described in Methods.

***Lolium perenne *sequence name**	***LoliumPerenne *SSR motif**	***Festuca arundinacea *accession no.**	***Festuca arundinacea *SSR motif**	***Brachypodium distachyon *accession no.**	***Brachypodium distachyon *SSR motif**	***Oryza sativa *accession no.**	***Oryza sativa *SSR motif**
gsa_002c_h11	(ACC)6	DT687024	(ACC)1AGC (ACC)2	BDEST01P1_Contig0330	No sequence at SSR motif	AK058436	No SSR motif
gsa_002d_g10	(CAG)4	DT696591	No SSR motif	BDEST01P1_Contig3728	No SSR motif	AK103926	No SSR motif
gsa_004b_a03	(GCG)4	DT706499	(GCG)4	BDEST01P1_Contig3390	No SSR motif	AK058218	No SSR motif
gsa_005a_e12	(CCG)4	DT703561	(CCG)4	BDEST01P1_Contig3040	(CCG)1	AK058256	(CCG)2CG (CCG)1
gsa_005c_d09	(GTC)4	DT706693	(GTC)4	BDEST01P1_Contig3222	No SSR motif	AK058745	No SSR motif
gsa_005d_h08	(CCG)4	DT680895	(CCG)1CA (CCG)1	BDEST01P1_Contig3684	No SSR motif	AK058262	(CCG)1C(CCG)1
gsa_006c_d05	(GCC)5	DT702323	(GCC)3	BDEST01P1_Contig3138	(GCC)2GGC (GCC)1	AK103918	(GCC)4
gsa_007c_g07	(TCC)4	DT679877	(TCC)2	BDEST01P1_Contig3812	(TCC)1	AK058319	No SSR motif
gsb_001a_g04	(TCC)4	DT693705	(TCC)4	BDEST01P1_Contig2531	(TCC)1CC (TCC)3	AK058266	(TCC)3
r_006d_e02	(CCG)4	DT714248	No sequence at SSR motif	BDEST01P1_Contig2672	(CCG)2TCG (CCG)4	AK058319	No SSR motif
rg1_005a_h06	(CTAT)4	DT703817	(CTAT)4	BDEST01P1_Contig3709	(CTAT)1	AK058206	(CTAT)1
rg1_010d_b12	(CCGA)4	DT711949	(CCGA)3	DV479746	No SSR motif	AK099825	(CCGA)1
rg3_008b_e10	(CCGA)4	DT696572	(CCGA)3	BDEST01P1_Contig3759	No SSR motif	AK099825	(CCGA)1
rg6_009d_f05	(GAT)4	DT704991	(GAT)4	BDEST01P1_ Contig3531	No sequence at SSR motif	AK073601	(GAT)3
sb_004a_b07	(GCA)4	DT681698	(GCA)1CGAGG (GCA)1	BDEST01P1_Contig3777	(GCA)2	AK058207	No SSR motif
ve_006d_h08	(CGC)4	DT714632	No sequence at SSR motif	DV488951	No sequence at SSR motif	AK071185	(CGC)2AGC (CGC)1
ve_007d_h07	(CAC)6	DT708139	No SSR motif	BDEST01P1_ Contig3106	(ACC)2GCCGGCC(ACC)1	AK103919	No SSR motif
vr_001c_h04	(CGC)4	DT685847	(CGC)1GCCC (CGC)1	BDEST01P1_ Contig0404	No sequence at SSR motif	AK058248	(CGC)8
vr_002a_c03	(TGG)4TGCTGCCC (CTG)4	CK802951	(TGG)4TGCTGCCC(CTG)4	BDEST01P1_ Contig3491	(TGG)1TGCTCCTGCTG(CTG)4	AK058240	(TGG)3TGCTCCAGTTG(CTG)4

n.d: No allelic sequence present in the EST collection.

In six of the 19 clusters (31%), there were no polymorphisms at the SSR motif between the sequences of the two closely related species *L. perenne *and *F. arundinacea*. The most frequent SSR motif polymorphisms between these two species were changes in the number of repeat units corresponding to 21% of the clusters. However, nucleotide substitutions, additions, and complete loss of SSR motifs were also observed (Table 6). None of the SSR motifs identified in *L. perenne *was completely conserved in *B. distachyon*. In six clusters (31%), the SSR motif was completely lost in *B. distachyon*, and in four clusters (21%) the *B. distachyon *SSR motif had fewer repeat units. In these four clusters, the *B. distachyon *SSR motif contained two to three fewer SSR motif units, compared to the corresponding *L. perenne *SSR motif. Nucleotide substitutions and additions were observed in five (26%) of the nineteen compared orthologous sequences (Table 6). None of the SSR motifs identified in *L. perenne *was completely conserved in *O. sativa*. In eight clusters (42%), the SSR motif was completely lost in *O. sativa*, and in six clusters the *O. sativa *SSR motif had fewer repeat units compared to the corresponding *L. perenne *SSR motif. However, in one cluster the *O. sativa *SSR motif had more repeat units compared to the corresponding *L. perenne *SSR motif (Table 6).

## Discussion

The present study was designed to create an SSR database of the transcribed region of the *L. perenne *genome by identification of SSRs in a dataset consisting of 25,744 ESTs from three different genotypes. Random sequencing of cDNA libraries leads to a high proportion of redundant ESTs. In this study, both the redundant and non-redundant dataset of EST-SSRs were included in the analysis. The redundant EST-SSRs were used to characterize the frequency of SSR motifs and to compare SSR motif polymorphisms between three genotypes of *L. perenne*, while the non-redundant dataset was used to characterize the type and distribution of EST-SSRs in the transcribed region of the *L. perenne *genome, and for a cross-species comparison of SSR polymorphisms within four species of the *Poaceae *family.

**Figure 2:**
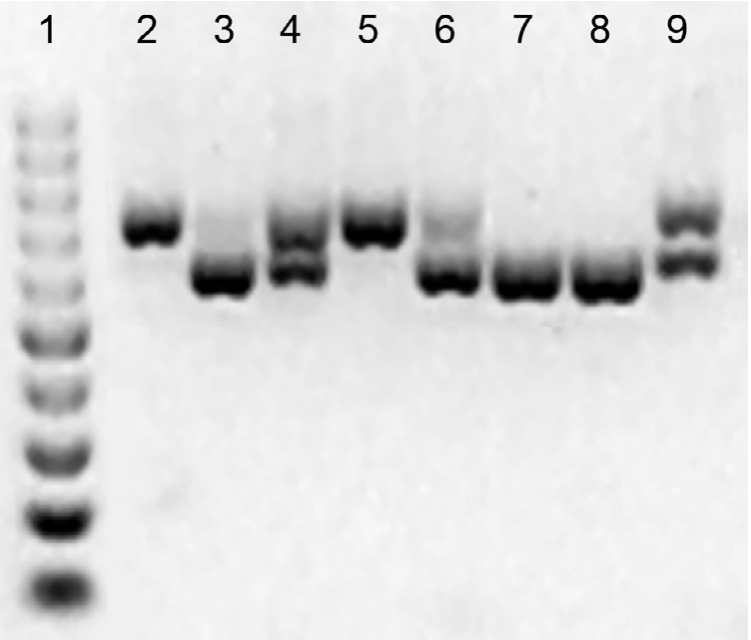
**PCR amplification of the microsatellite (CGA)4 within the EST-clone ve_002b_h12 in eight selected and representative *Lolium perenne *F2 genotypes of the VrnA mapping population [6]. **Lane 1: 100 bp ladder DNA-marker; lane 2: NV#20/30-39/008; lane 3: NV#20/30-39/018; lane 4: NV#20/30-39/091; lane 5: NV#20/30-39/102; lane 6: NV#20/30-39/119; lane 7: NV#20/30-39/224; lane 8: NV#20/30-39/392; lane 9: NV#20/30-39/438. The primers used were G05_132_L1 (CAGATGCGCATGTCCTACAG) and G05_132_R1 (CTTGCTCTTGTCCGAATCGT). PCR and electrophoresis was performed as described previously [6].

A total number of 1,458 redundant and 955 non-redundant SSRs were identified, corresponding to 5.66 and 3.71% of redundant and non-redundant ESTs, respectively. Preliminary results exemplified in Figure 2 indicate that some of the EST-SSRs identified in this study are polymorphic in the mapping population VrnA [6] and, thus, can be used for marker development, demonstrating that *L. perenne *ESTs are a valuable resource for SSR marker development. The transcribed region of the genome of the genotype F6 contains a significantly higher frequency of SSRs. Approximately 10% of the ESTs from the genotype F6 contain an SSR, compared to approximately 3.6% in the other two genotypes, indicating a large genotypic variation in the frequency of SSR motifs. To our knowledge, this is the first report where the frequency of SSRs in ESTs from different genotypes within one plant species has been compared. The results suggest that it would be reasonable to generate a small number of ESTs from different genotypes, to decide which one is the best for EST-SSR development.

However, the differences observed in the frequencies of SSR motifs might not only be genotypic differences, but also be due to different cDNA libraries established for the three genotypes, because the composition of expressed genes is likely differing between the thirteen cDNA libraries selected for EST development. NV#20F1-30 and NV#20F1-39 are full-sibs [6], and most of the differences in SSR motif frequencies between these two genotypes can, therefore, be attributed to differentially expressed genes in the different cDNA libraries selected for EST development. Comparing the frequencies of SSR motifs in ESTs developed from four cDNA libraries of NV#20F1-30 with three libraries of NV#20F1-39 revealed no significant differences in frequencies of SSR motifs between these two genotypes. Thus, the variation in the frequency of SSR motifs can most likely be attributed to genotypic differences between F6, and NV#20F1-30 and NV#20F1-39. However, because most of the NV#20F1-30 and NV#20F1-39 ESTs are from leaf cDNA libraries, whereas the majority of ESTs from F6 comes from a root cDNA library, still the possibility cannot be ruled out completely, that the root cDNA library and other cDNA libraries prepared from the genotype F6 contains more SSRs.

The average frequency of 3.71% non-redundant SSRs in the transcribed region of the *L. perenne *genome is within the same range as previously reported for other plant species [14,23,41-43]. However, caution should be exerted when SSRs frequencies are compared between different plant species, because of differences in the SSR search parameters.

Approximately 96% of all SSRs analysed were shorter than 21 bp, indicating that the length of SSR motifs in the transcribed region of the *L. perenne *genome are size-restricted. In addition, 6 bp di-repeats comprise 40 to 64% of the di-repeats in the three genotypes, indicating that di-repeats, which do not perturb the open reading frame are preferred over others. The expansion of SSR repeats in transcribed regions of the genome is limited by functional and evolutionary constraints [44,45], because longer repeats have higher mutation rates and are, thus, less stable [20,46]. Short SSRs are probably generated by random mutations and then expanded by DNA polymerase slippage. Thus, the base composition of a sequence that precedes the evolution of SSRs is expected to influence SSR density [47,48]. The higher frequency of SSRs in the transcribed region of the genotype F6 could indicate, that the genome of this genotype is more prone to mutations and/or DNA polymerase slippage compared to the genome of the other two genotypes. This indicates that there might be genotype specific cellular factors that interact with SSR motifs and play an important role in generating short tandem repeats [49].

Previous studies have shown that tri-nucleotide repeats predominate in coding regions of plant genomes [12,50], as well as in other genomes of higher eukaryotic organisms [45,51,52], because expansions or deletions in coding regions can be tolerated for tri- and hexa-nucleotide unit repeats, which do not perturb reading frames [53]. In *L. perenne*, the most common SSR repeat units were also found to be tri-nucleotide repeats, constituting between 59 and 85% of the repeats in the three genotypes included in this study, while di- and tetra-nucleotide units constitute the majority of the remaining motifs. Only a few penta- and hexa-nucleotide repeat units were identified. A wide variety of tri-nucleotide repeat units were represented at high percentages, however, the abundance of the different types of repeat units differed, especially between the genotype F6 and the two other genotypes. The repeat motif (CCG/CGG)*n *was highly represented in 42% of EST-SSRs from the genotype F6, while it was represented at a low frequency of approximately 1% in the other two genotypes.

In the two genotypes NV#20F1-30 and NV#20F1-39 the most abundant repeat encodes for the amino acid threonine, while the most abundant repeat in the genotype F6 encodes for the amino acid proline. Analysis of all protein sequences from the SWISS-PROT database for single amino acid repeats, tandem oligo-peptide repeats, and periodically conserved amino acids showed that repeats of glutamine, serine, glutamic acid, glycine and alanine seems to be fairly well tolerated in many proteins [54]. Of these amino acids, only the amino acid serine were found in the tri-nucleotide repeats of *L. perenne*, while the other amino acid residues were not represented. The presence of SSRs in transcripts of genes suggests that they may have a role in gene expression or function. In *O. sativa*, the length of a poly(CT) SSR in the 5'-untranslated region of the *waxy *gene is associated with amylose content [55], and in *Z. mays *a SSR the 5'-untranslated region of some ribosomal genes, have been suggested to be involved in the regulation of fertilization [56].

A total of 22 contigs containing EST sequences with either allelic- and/or genotypic SSR polymorphisms were identified, corresponding to 2.3% of the non-redundant EST-SSR contigs. The remaining 499 contigs (97.7%) contained no SSR motif polymorphism, indicating a selection against length polymorphisms in the transcribed region of the *L. perenne *genome. In all contigs containing an SSR motif polymorphism, the polymorphisms identified were changes in the number of repeat units, while no contigs were identified with changes in the repeat type or complete loss of the SSR motif. The majority of the SSR polymorphisms were allelic polymorphisms, and most of the SSR motif polymorphisms were one to two repeat unit changes. All polymorphisms identified, except for polymorphisms in compound SSRs, were changes in the number of repeat units, while no single nucleotide additions or deletions were identified, that otherwise would perturb the open reading frame.

Several studies have shown that SSRs developed for one species could be used in related plant species, and that the success of cross-species amplification depends on the evolutionary relatedness [57]. The availability of the *O. sativa *genome sequence provides a rich source of molecular information [58]. On the contrary, this type of information is limited for most forage and turf grass species. Comparative mapping can make use of the genomic information available for *O. sativa *by applying this knowledge to less studied forage and turf species.

The transferability of the *L. perenne *SSR markers between species of the *Poaceae *family were performed *in silico*, to evaluate if the SSRs can be used as anchor markers for comparative mapping and evolutionary studies. SSRs designed from EST sequences are especially valuable owing to their genome location, which implies constraints on length, motif, abundance and flanking regions, the latter of particular interest in this context, because common primers can be designed to conserved flanking regions. However, before primers are designed it is necessary to evaluate if the SSR motif is conserved between related species, and therefore useful for SSR marker development. Blast searches using the 955 non-redundant *Lolium perenne *EST-SSRs as query sequences against 41,834 *F. arundinacea *EST sequences, 3,818 *B. distachyon *contigs, and 32,132 full-length *O. sativa *cDNA sequences resulted in 833, 540, and 26 orthologous sequences, respectively. However, because the amount of sequence information available differs between the species included in this study, the number of hits cannot be directly compared. A total of 19 clusters were identified containing sequences of all four species. Analysis of the clusters indicates that the SSR motif in general is conserved in the closely related species *F. arundinacea *apart from differences in the length of the SSR motif. In contrast, the SSR motif is often lost in the more distant related species *B. distachyon *and *O. sativa*.

In a previous study, the transferability of genomic SSR markers developed for *F. arundinacea *across multiple grass species was investigated [59]. A total of 511 *F. arundinacea *genomic SSRs were used to screen the six species; *F. arundinacea*,*F. arundinacea *var. *Glaucescens *(tetraploid), *F. pratensis*, *L. perenne*, *O. sativa*, and *Triticum aestivum*, representing three tribes and two subfamilies of the *Poaceae *family. Most SSRs could be amplified in all forage and turf grasses but not in cereal species included in that study [59]. These results support the results presented in this study, where SSR motifs are more conserved between *L. perenne *and *F. arundinacea*, compared to *B. distachyon*, and *O. sativa*.

Experimental validation of these hypothetical transferable SSRs and their polymorphism is needed, to validate the results of the *in silico *analysis of SSR motif polymorphisms between the species included in this study. However, the *in silico *analysis of the conservation of SSR motifs across species is a valuable tool, because it gives an indication of how distant related species can be, when experiments for comparative mapping and evolutionary studies are designed. Furthermore, the results are valuable for estimating how large the chance is, to find SSR motifs as prerequisite for a polymorphic marker, in closely- as well as distant related species.

With the *L. perenne *EST-SSRs presented in this paper, a valuable tool has been developed for further genetic-, genomic-, and plant breeding applications on the intra- as well as on the inter-species level.

## Conclusion

In this study, we present a comprehensive set of publicly available EST-derived SSRs from three genotypes of *Lolium perenne*, one of the major grass species used for turf and forage in the temperate regions.

A total of 955 non-redundant SSRs were detected *in silico *using clustered and assembled EST data. Tri-nucleotide repeats were the most abundant type of repeats followed by di- and tetra-nucleotide repeats. Approximately 96% of all SSRs identified were shorter than 21 bp, indicating that the length of SSR motifs in the transcribed region of the *L. perenne *genome are size-restricted.

A large variation in the number of SSRs in transcribed regions of the three genotypes was observed, ranging from one SSR per 10.9 kb in genotype NV#20F1-30 to one SSR per 2.7 kb in the genotype F6. This result suggests that several genotypes should be screened to find the best genotype for SSR discovery in transcribed sequences.

All allelic SSR polymorphisms identified within *L. perenne *were changes in the number of repeat units. When comparing SSR motifs from *L. perenne *to SSR motifs in orthologous sequences from *F. arundinacea*, *B. distachyon*, and *O. sativa *changes both in the number of repeats, and complete loss of the SSR motifs were observed. Comparing orthologous sequences of *L. perenne *and *F. arundinacea *revealed that the most frequent SSR motif polymorphisms between these two species were changes in the number of repeat units corresponding to 21% of the clusters, while there were no SSR polymorphisms in 31% of the analysed clusters. Thus, the EST-SSRs are suitable for synteny studies between these two species.

In contrast, none of the SSR motifs identified in *L. perenne *was completely conserved in the more distant related species *B. distachyon *and *O. sativa*. In 31% of the clusters the SSR motif was completely lost in *B. distachyon*, and in 21% the SSR motif had fewer repeat units. This suggests that the EST-SSRs are less suitable for synteny studies outside the *Lolium*/*Festuca *complex.

With the EST-SSR set, a valuable tool has been made publicly available for numerous further genetic and genomic applications on intra- and inter-species level.

## Methods

### Library construction and DNA sequencing

Thirteen directional cDNA libraries were constructed from a range of tissues and developmental stages (Table 1). Tissues were obtained from three different *L. perenne *genotypes: NV#20F1-30, NV#20F1-39 [6], and F6 (DLF-Trifolium Ltd.). The two genotypes NV#20F1-30 and NV#20F1-39 are F1 offspring (full-sibs) of a cross between two genotypes from the variety Veyo and the ecotype Falster, respectively, and have thus the same heterozygous parents [6].

RNA was isolated using Tri^® ^Reagent (Sigma-Aldrich, St. Louis, MO, USA), and the cDNA libraries were constructed using the Creator™ SMART™ cDNA Library Construction Kit (BD Biosciences, Palo Alto, CA, USA), according to the manufacturer's instructions. The cDNAs were cloned directionally into the asymmetric *SfiI *sites of the pDNR-LIB vector, transformed into electrocompetent DH10B T1-phage-resistant *Escherichia coli *cells (Invitrogen, Carlsbad, CA, USA), and robotically arrayed into 384-well plates. A total of 31,379 random clones were subjected to single-pass sequencing reactions from the 5'end using BigDye^® ^Terminator v3.1 sequencing chemistry and analyzed on an ABI Prism 3700 DNA Analyzer (Applied Biosystems, Foster City, CA, USA). Colony picking and sequencing was performed by MWG Biotech (MWG Biotech, Ebersberg, Germany). Base calling, vector trimming, removal of low quality bases, and clustering and assembly of the ESTs were performed using the PHRED and PHRAP/CROSS_MATCH software packages [60-62]. Sequences with less than 100 PHRED ≥ 20 quality bases after trimming were discarded. A complete description of the cDNA library construction methods will be reported elsewhere.

### EST database and identification of EST-SSRs

An EST database was developed consisting of 25,744 ESTs corresponding to 8.53 Mb of sequence (Asp et al. unpublished). Protein functions were predicted by BlastX similarity searches against the protein database in the GenBank [63], and annotated in terms of the associated biological processes, cellular components, and molecular functions using the Gene Ontology vocabulary.

The Perl script MIcroSAtelitte (MISA) [28] was used to identify SSRs in the *L. perenne *EST sequences. The parameters for the SSR search were defined as follows. The size of motifs was two to six nucleotides, and the minimum repeat unit was defined as six for di-nucleotides and four for tri-, tetra-, penta-, and hexa-nucleotides. Compound SSRs were defined as ≥ 2 SSRs interrupted by ≤ 50 bases.

### Allelic and genotypic SSR motif polymorphism analysis

*L. perenne *is a diploid (2n = 2x = 14) outbreeding species with self-incompatibility being controlled by two genetic loci. A maximum number of two alleles can therefore be expected in each genotype. The 3,195 *L. perenne *contigs was queried using MISA to identify SSR containing contigs. The individual sequences within each SSR containing contig was subsequently analysed for SSRs using MISA to identify allelic and/or genotypic SSR motif polymorphisms.

### Cross-species SSR motif polymorphism analysis

The cross-species SSR motif polymorphism analysis was performed by comparing orthologous sequences of *L. perenne*, *F. arundinacea, O. sativa*, and *B. distachyon*. A total of 41,834 *F. arundinacea *ESTs were downloaded from dbEST in the GenBank [64], 32,132 *O. sativa *full-length sequences were downloaded from KOME [65], and 3,818 *B. distachyon *contigs were downloaded from the Genomics and Gene Discovery bEST Resource home page [66]. The sequences were subsequently blasted (e-value 1.00E-10) using BlastN against 1,458 *L. perenne *ESTs containing SSRs, to identify the orthologous sequences. A relational database was created and used to store all information related to the DNA sequences of the four species, including DNA sequences, similarity search results, query search results, SSR presence, SSR motif type, and SSR locus polymorphisms between the four species included in this study.

### Data access

Sequences described have been submitted to GenBank. Submitted sequences are in the accession number range of ES699013 to ES700454.

## Authors' contributions

TA and TD constructed the cDNA libraries for EST sequencing. TA and UKF conducted the bioinformatic analysis. TA, KKN, and TL designed and coordinated the study. TA interpreted the data, performed the statistical analysis, and drafted the manuscript. TL assisted in drafting the manuscript. All authors read and approved the final manuscript.
